# TRIM28 contributes to epidermal homeostasis and is associated with altered p53 regulation

**DOI:** 10.3389/fcell.2026.1903169

**Published:** 2026-09-08

**Authors:** Lucian Beer, Hannes Kühtreiber, Lisa Auer, Tanja Wagner, Bahar Golabi, Sophie Alexandra Röder, Melanie Salek, Michael Mildner

**Affiliations:** 1 Department of Biomedical Imaging and Image-guided Therapy, Medical University of Vienna, Vienna, Austria; 2 Department of Dermatology, Medical University of Vienna, Vienna, Austria; 3 Department of Thoracic Surgery, Medical University of Vienna, Vienna, Austria; 4 Aposcience AG, Vienna, Austria; 5 Division of Neurophysiology and Neuropharmacology, Centre of Physiology and Pharmacology, Medical University of Vienna, Vienna, Austria

**Keywords:** E3 ubiquitin ligase, epidermal differentiation, KAP1, keratinocyte, p53, skin equivalent, TRIM28, ubiquitination

## Abstract

Epidermal keratinocytes (KCs) undergo a tightly orchestrated differentiation programme that culminates in the formation of a protective barrier between the body and the environment. Although this process has been studied extensively, several regulators that coordinate KC survival, stratification, and terminal differentiation remain incompletely defined. To identify mechanisms governing epidermal homeostasis, we compared transcriptomic profiles of undifferentiated KCs, KCs differentiated in monolayer culture, and fully stratified KCs in three-dimensional organotypic skin equivalents. Both differentiation models showed downregulation of cell-cycle-associated transcripts and induction of KC differentiation genes, whereas genes involved in extracellular-matrix organisation were preferentially induced in skin equivalents. Transcription-factor activity inference based on target gene expression identified the transcriptional repressor and ubiquitin/SUMO E3 ligase TRIM28 as a candidate regulator of epidermal homeostasis. Single-cell RNA sequencing and immunostaining of human epidermis revealed strong TRIM28 expression throughout the living epidermal layers, with marked loss in the outermost terminally differentiated compartment. Functional depletion of TRIM28 in primary human KCs impaired epidermal development, increased apoptosis, and produced markedly thinner organotypic epidermis despite preserved stratification. TRIM28-deficient skin equivalents showed limited transcriptional reprogramming associated with reduced p53 ubiquitination, p53 accumulation, and apoptosis of basal KCs. Collectively, these findings identify TRIM28 as an important candidate regulator of epidermal homeostasis and suggest that TRIM28 supports epidermal expansion, at least in part, by contributing to p53 turnover.

## 1 Introduction

The human epidermis forms a highly specialised barrier that protects the body from environmental stressors while preventing excessive water loss. This barrier is maintained by a stratified squamous epithelium in which proliferative keratinocytes (KCs) are confined mainly to the basal layer, whereas suprabasal KCs progressively withdraw from the cell cycle, initiate terminal differentiation, and ultimately form the cornified layer. The transition from proliferation to differentiation requires tightly coordinated changes in gene expression, protein turnover, and cellular architecture. Although many signalling pathways and transcription factors (TFs) have been implicated in this process, the mechanisms that integrate KC survival, stratification, and terminal differentiation remain incompletely understood (Wikramanayake et al., 2014; Gęgotek and Skrzydlewska, 2015).

One central regulator of epidermal growth and stress responses is the tumour-suppressor protein p53. In the skin, p53 contributes to epidermal development, tumour suppression, wound repair, and responses to genotoxic stress (Botchkarev and Flores, 2014; Freije et al., 2014). Its canonical functions include the regulation of cell-cycle progression, DNA repair, apoptosis, and chromosomal integrity, and p53-dependent transcriptional programmes influence processes ranging from cell-cycle control to signalling and metabolism (Böhlig and Rother, 2011; Liao et al., 2016). Importantly, p53 is required not only in damaged or stressed epidermis but also during normal epidermal development under homeostatic conditions (Einspahr et al., 1999; Cai et al., 2012). During KC differentiation, p53 expression decreases whereas p53 activity increases, a shift that contributes to the repression of keratin 14 and the induction of differentiation-associated programmes (Einspahr et al., 1999; Cai et al., 2012). These observations suggest that epidermal homeostasis depends on precise spatial and temporal control of p53 abundance and activity: insufficient p53 signalling may disturb differentiation, whereas excessive or misplaced p53 activity can compromise proliferative-cell survival.

Tripartite motif-containing 28 (TRIM28), also known as Krüppel-associated box (KRAB)-associated protein 1 (KAP1), is a multifunctional nuclear protein with established roles in transcriptional repression, chromatin regulation, and post-translational protein control. Structurally, TRIM28 contains several conserved domains, including a RING finger, B-boxes, and leucine-zipper α-helical coiled-coil regions (Wang et al., 2005). It belongs to the TRIM protein family, several members of which function as E3 ligases that modify substrate proteins by ubiquitination or related post-translational mechanisms. For example, TRIM21 promotes degradation of interferon regulatory factor 3 (IRF3) (Higgs et al., 2008), TRIM25 catalyses K63-linked ubiquitination of the RNA sensor RIG-I (Gack et al., 2007), and TRIM30α regulates the ubiquitination and degradation of the TGF-β-activated kinase 1 (MAP3K7)-binding proteins TAB2 and TAB3 (Shi et al., 2008). TRIM28 itself has small ubiquitin-related modifier (SUMO) E3 ligase activity (Liang et al., 2011) and interacts with the p53-mouse double minute two homolog (MDM2) axis through its N-terminal coiled-coil domain and the central acidic domain of MDM2, thereby promoting proteasomal degradation of p53 (Wang et al., 2005; Doyle et al., 2010). TRIM28 has also been reported to ubiquitinate BCL2A1, an anti-apoptotic member of the BCL-2 family, thereby influencing cell survival (Lionnard et al., 2019). Consistent with these diverse activities, TRIM28 is frequently dysregulated in malignancies, including non-small-cell lung cancer and breast cancer, where it has been linked to tumour growth and metastasis (Liu et al., 2013; Wei et al., 2016).

Despite this broad functional repertoire, the role of TRIM28 in epidermal differentiation and tissue homeostasis remains poorly defined. In particular, it is unclear whether TRIM28 mainly acts as a transcriptional regulator during KC differentiation or whether its post-translational functions contribute to epidermal organisation by controlling survival pathways such as p53 turnover. We therefore hypothesised that TRIM28 supports epidermal homeostasis by coordinating KC survival and stratification during human skin development. To test this hypothesis, we combined transcriptomic profiling of monolayer and organotypic differentiation models, transcription-factor activity inference, single-cell RNA sequencing, immunostaining of human epidermis, and siRNA-mediated loss-of-function experiments in organotypic skin equivalents. This integrated approach allowed us to define the spatial regulation of TRIM28 during KC differentiation and to assess its functional contribution to epidermal architecture.

## 2 Materials and methods

### 2.1 Ethics statement

The use of resected skin tissue from healthy donors undergoing elective surgery was approved by the ethics committee of the Medical University of Vienna (EK vote 217/2010) in accordance with the Declaration of Helsinki and the guidelines of the Council for International Organizations of Medical Sciences (CIOMS). Written informed consent was obtained from all donors.

### 2.2 Cell culture and siRNA transfection

Primary human KCs were cultured as described previously (Mildner et al., 2006). Three siRNAs specific for TRIM28, each used separately, and a scrambled control were obtained from Fisher Scientific (Waltham, MA, USA; HSS115468, HSS115469, HSS115470). For transfection, third-passage KCs at 50%–60% confluence were transfected with Lipofectamine 2000 (Fisher Scientific) according to a published protocol (Mildner et al., 2010). Briefly, 5 mL OPTI-MEM medium (Fisher Scientific) was mixed with 50 µL Lipofectamine 2000 and 130 µL of a 20 µM siRNA or scrambled control RNA (Fisher Scientific). After incubation at room temperature for 20 min, the solution was added to 20 mL of antibiotic-free KGM-2 medium (Lonza, Basel, Switzerland), applied to the KC cultures, and incubated for 24 h.

### 2.3 Preparation of organotypic knock-down skin cultures

*In vitro* skin cultures were established as previously described (Mildner et al., 2006; Mildner et al., 2010). Briefly, a suspension of type I collagen (Vitrogen 100, Collagen Corporation, CA, USA) containing 1 × 10^5^ fibroblasts per ml was poured into cell-culture inserts (3 µm pore size; BD Bioscience, Bedford, MA, USA) and allowed to gel for 2 h at 37 °C in a humidified atmosphere. The gels were equilibrated with KGM-2 for 2 h, after which 1.5 × 10^6^ KCs, either non-transfected or transfected with the respective siRNAs (Thermo Fisher), in a total volume of 2 mL KGM-2 were seeded onto the collagen gel. After overnight incubation, the medium was removed from both the inserts and the external wells and replaced in the external wells with serum-free KC defined medium (SKDM), consisting of KGM-2 without bovine pituitary extract supplemented with 1.3 mM calcium (Sigma, Vienna, Austria), 50 μg/mL ascorbic acid (Sigma), 10 μg/mL transferrin (Sigma), and 0.1% bovine serum albumin (Sigma). Cultures were maintained at the air–liquid interface for up to 7 days; unless stated otherwise, skin equivalents were harvested on day 7 after exposure to the air–liquid interface.

### 2.4 RNA isolation and cDNA synthesis

RNA isolation and cDNA synthesis were performed as described previously (Beer et al., 2015). For skin equivalents, the collagen–fibroblast dermal matrix was removed prior to RNA extraction. Total RNA was therefore isolated exclusively from the epidermal compartment. Total RNA from KCs and skin equivalents was prepared using TRIzol reagent according to the manufacturer’s instructions (Thermo Fisher). The concentration and A260/280 ratio of purified RNA were measured with a NanoDrop ND-1000 spectrophotometer (Thermo Scientific, Wilmington, DE, USA), and the quality of selected samples was assessed with an Agilent 2100 Bioanalyzer (Agilent Technologies, Santa Clara, CA, USA). Samples were stored at −80 °C until further use.

### 2.5 Microarray expression data analysis

All samples used for microarray analysis had an RNA integrity score between 8.5 and 10; 150 ng of total RNA, without the small-RNA fraction, was analysed, and all steps were performed according to the manufacturer’s protocol. Two independent Affymetrix datasets were analysed. The differentiation dataset (proliferating KCs, monolayer-differentiated KCs and skin equivalents; three biological replicates per condition, GEO accession GSE145059) was described previously (Beer et al., 2020). The TRIM28 knock-down dataset comprised paired scrambled-control and TRIM28-siRNA samples from three donors. Computational analysis of the raw microarray data (.cel files) was performed in R (version 4.3.1; The R Foundation, Vienna, Austria) and RStudio (version 2023.6.2.0 + 561). Raw data (.cel files) were preprocessed with the Robust Multiarray Average (RMA) method in the oligo package (version 1.64.1). Probe sets were annotated with gene symbols using the hugene20sttranscriptcluster.db package (version 8.8.0); only probe IDs with a valid annotation were retained, and probe sets mapping to the same gene symbol were averaged. Differential expression was assessed with limma (version 3.56.2) (Ritchie et al., 2015) using a design matrix without intercept and contrasts for all pairwise comparisons between the three conditions; p-values were adjusted by the Benjamini–Hochberg procedure. Principal component analysis (PCA) was performed on the normalised expression data after filtering out genes with zero variance across samples. Differentially expressed genes (DEGs) were defined as |logFC| ≥ 1 with a false-discovery rate (FDR) < 0.05. Area-proportional Euler diagrams of the up- and downregulated gene sets were drawn with the eulerr package (version 7.0.0), heatmaps with pheatmap (version 1.0.12), and volcano plots with EnhancedVolcano (version 1.18.0) (Blighe et al., 2023). Heatmaps were generated with ComplexHeatmap (version 2.16.0) (Gu et al., 2016).

### 2.6 Transcription-factor activity inference

Regulator activity was inferred from the expression of curated target genes with the decoupleR package (version 2.6.0) (Badia-I-Mompel et al., 2022), applying a univariate linear model (ULM) to log2-transformed expression values. The prior-knowledge network was the CollecTRI regulon collection (Müller-Dott et al., 2023), retrieved through OmnipathR (version 3.8.2); regulons with fewer than five targets were excluded. This approach scores a regulator by the coordinated expression of its documented targets rather than by its own transcript abundance or by sequence motifs, and it therefore also covers non-classical, non-sequence-specific regulators such as TRIM28, which is annotated in CollecTRI as a transcriptional repressor. The regulators with the highest variance in activity score across all samples, together with TRIM28, were retained; scores were averaged per condition, scaled across conditions, and visualised with the pheatmap package (version 1.0.12).

### 2.7 Functional annotation and pathway analysis

Up- and downregulated gene lists (|logFC| ≥ 1, nominal p < 0.05) were submitted separately to Enrichr (Kuleshov et al., 2016) through the enrichR package (version 3.2) and tested against the Reactome_2022 gene-set library. Terms with p < 0.05 were ranked by the Enrichr combined score, which integrates the Fisher’s exact-test p-value and the deviation of the observed rank from the expected rank, and the highest-scoring terms per gene list were displayed as dot plots showing gene ratio, combined score, and −log10 (p-value). Visualisation was performed with the ggplot2 package (version 3.4.2) (Wickham, 2016).

### 2.8 Single-cell sample preparation and sequencing

Skin samples were obtained from three healthy adult donors undergoing abdominoplasty, comprising one man aged 54 years, one man aged 23 years, and one woman aged 29 years. Six biopsies were prepared from each sample and digested with Dispase II overnight at 4 °C to separate the epidermis from the dermis. One-half of the separated epidermis was dissociated with the GentleMACS Human Whole Skin dissociation kit (Miltenyi Biotec, Bergisch Gladbach, Germany) for 2.5 h at 37 °C according to the manufacturer’s instructions, whereas the other half was digested with 0.05% Trypsin-EDTA and DNase I for 7 min at 37 °C. The two cell suspensions were pooled, processed on the GentleMACS OctoDissociator (Miltenyi Biotec), and passed sequentially through 100 μm and 40 μm cell strainers. Cells were stained with DAPI for 10 s, resuspended in 0.04% BSA in PBS, and washed twice before viable cells were isolated by fluorescence-activated cell sorting on an AriaFusion cell sorter (BD Biosciences, San Jose, CA, USA).

Single-cell gene expression libraries were generated on the 10x Genomics Chromium platform (10x Genomics, Pleasanton, CA, USA) using Single Cell 3′v2 chemistry. Immediately after sorting, the cell suspensions were loaded onto the Chromium instrument, and Gel Bead-in-Emulsion formation, complementary DNA synthesis, library preparation, and sequencing were performed by the Biomedical Sequencing Core Facility of the Center for Molecular Medicine (Vienna, Austria). Libraries were sequenced on an Illumina HiSeq 3,000/4,000 instrument (Illumina, San Diego, CA, USA), yielding transcriptomes from approximately 29,000 cells across the three donors. Raw sequencing reads were demultiplexed with the 10x Genomics Cell Ranger fastq pipeline and aligned to the human GRCh38 reference genome with the Cell Ranger count pipeline, which quantified cell barcodes and unique molecular identifiers to produce a gene expression matrix for each donor that served as input for the analysis described below.

### 2.9 Single-cell RNA-sequencing analysis

scRNA-seq analysis was conducted in R using Seurat (version 5; Hao et al., 2024) with a fixed random seed (1,234). Count matrices were imported per 10x capture, retaining genes detected in at least three cells. For quality control, the percentages of mitochondrial, ribosomal and haemoglobin transcripts and the numbers of unique molecular identifiers (UMIs) and detected genes were calculated. The same cutoffs were applied to all three samples: cells were retained with 800–4,000 detected genes, 2,000–25,000 UMIs and below 10% mitochondrial transcripts. Doublets were then identified per capture with scDblFinder (Germain et al., 2021) using random artificial doublet generation, and predicted doublets were removed; the resulting quality-control distributions, doublet calls and integration diagnostics are shown in Supplementary Figure S1. The filtered samples were merged and log-normalised (scale factor 10,000), the 3,000 most variable features were selected by variance-stabilising transformation (VST), and the data were scaled. Fifty principal components were computed and the first 30 were used for all downstream steps. Donor effects were corrected by reciprocal PCA integration (RPCAIntegration, k. anchor = 20). A shared-nearest-neighbour graph was built on the integrated embedding, Louvain clustering was performed at resolutions from 0.2 to 1.0, and resolution 0.4 was selected for the final annotation. Two-dimensional visualisation used Uniform Manifold Approximation and Projection (UMAP). Cluster markers were determined with a Wilcoxon rank-sum test, and the resulting eight clusters were annotated manually using canonical epidermal markers (basal: KRT5, KRT14, KRT15, TP63, ITGA6; cycling: MKI67, TOP2A, PCNA; suprabasal and differentiated: KRT1, KRT10, KRT2, IVL, FLG, LOR; melanocytes: PMEL, MLANA, TYR; immune cells: PTPRC, CD207, CD3D), yielding six KC populations, melanocytes and immune cells. TRIM28 expression across the annotated KC populations was visualised as violin plots of log-normalised expression.

### 2.10 Re-analysis of published single-cell datasets

To assess TRIM28 expression in inflammatory and neoplastic skin, publicly available human single-cell RNA-sequencing datasets were re-processed with the same Seurat workflow and as previously published (Kühtreiber et al., 2025): psoriasis (lesional, non-lesional and healthy skin; GSE173706; Ma et al., 2023), atopic dermatitis (GSE153760; Bangert et al., 2021) and keratinocyte tumours (actinic keratosis, squamous cell carcinoma *in situ* and cutaneous squamous cell carcinoma with matched healthy skin; GSE218170, GSE193304 and GSE144236; Schütz et al., 2023; Zou et al., 2023; Ji et al., 2020). KCs were annotated into proliferating (pKC), basal (bKC), suprabasal (sbKC) and differentiated (dKC) populations using the marker panel above, and TRIM28 expression per population and condition was displayed as dot plots of average expression and percentage of expressing cells (Supplementary Figure S2).

### 2.11 Quantitative real-time PCR (qPCR)

qPCR was performed with the LightCycler FastStart DNA Master SYBR Green I kit (Roche Applied Science, Penzberg, Germany) on a LightCycler 480 instrument (Roche Applied Science) as described previously (Beer et al., 2014; Beer et al., 2020). Primers were designed with Primer3 (http://primer3.ut.ee/) and synthesised by Microsynth AG (Vienna, Austria). β2-microglobulin (B2M) was used as the reference gene. It was selected after evaluation of several housekeeping genes, as it showed the most stable expression across all experimental conditions tested. Comparable results were obtained using ALAS1 as an alternative reference gene (data not shown).

### 2.12 SDS-PAGE and western blot

For Western blot analysis, KCs and skin models were lysed in SDS-PAGE loading buffer, sonicated, and centrifuged. Sodium dodecyl sulfate polyacrylamide gel electrophoresis (SDS-PAGE) was performed on 8%–18% gradient gels (Sigma) under reducing conditions. Proteins were electro-transferred onto nitrocellulose membranes (Bio-Rad, Hercules, CA, USA) and immunodetected with primary antibodies against TRIM28 (1 μg/mL; Abcam, Cambridge, United Kingdom), ubiquitin (1:1,000; Sigma-Aldrich), and GAPDH (1:400; Biogenesis, Poole, United Kingdom). Where indicated, total protein staining of the membrane was used as the loading control instead of GAPDH. Band intensities were quantified densitometrically in ImageJ (Schneider et al., 2012) and normalised to the corresponding loading control. Peroxidase-conjugated secondary antibodies were detected by chemiluminescence using the ChemiGlow reagent (Biozyme Laboratories Limited, South Wales, United Kingdom) according to the manufacturer’s instructions.

### 2.13 Immunofluorescence staining and image quantification

Immunofluorescence labelling was conducted on 5 µm sections of formalin-fixed, paraffin-embedded human skin and organotypic skin equivalents according to routine laboratory protocols. Following deparaffinisation and rehydration, sections were subjected to antigen retrieval by heating in a microwave oven at 500 W for 5 min in Target Retrieval Solution (Dako, Glostrup, Denmark). After blocking, sections were incubated overnight at 4 °C in PBS containing 2% BSA with primary antibodies against p53 (2 μg/mL, Sigma-Aldrich, Vienna, Austria), active caspase-3 (1 μg/mL, BD Pharmingen, San Jose, CA, USA), TRIM28 (1 μg/mL; Abcam, Cambridge, United Kingdom), keratin 10 (1 μg/mL), loricrin (1 μg/mL), filaggrin (1 μg/mL) and involucrin (0.2 μg/mL, all BioLegend, San Diego, CA, USA). After washing, sections were incubated with the corresponding fluorophore-conjugated secondary antibody and counterstained with DAPI. Sections were mounted in aqueous mounting medium and imaged on an Olympus BX63 microscope (Olympus, Tokyo, Japan) with Olympus CellSens Dimension v2.3 software, using standardised exposure times for all samples of a given staining. Signal quantification was performed in ImageJ (Schneider et al., 2012) on unmodified images with identical settings for all conditions. Nuclear TRIM28 intensity was measured in human skin from four independent donors. Individual nuclei were assigned to the basal, suprabasal or last living differentiated layer, and the nuclei of each layer were averaged per donor and layer, which served as the unit for statistical inference. Differentiation-marker signals were expressed as mean fluorescence intensity of the epidermal compartment, and p53-and active-caspase-3-positive cells were counted per section and normalised to the corresponding control. In each case the values from all sections of one skin equivalent were averaged to the mean value per skin equivalent before analysis. Epidermal thickness was measured perpendicular to the dermal-epidermal junction at defined intervals along each section of hematoxylin-eosin-stained skin equivalents.

### 2.14 Cell-cycle analysis

Cell-cycle analysis was performed with the BrdU Flow Kit (BD Biosciences, Franklin Lakes, NJ, USA) according to the manufacturer’s instructions. Briefly, 2 and 7 days after siRNA transfection, cells were incubated with BrdU (10 µM) for 4 h; S-phase and apoptotic fractions were determined in three independent experiments. After fixation with 100 µL BD Cytofix/Cytoperm buffer for 15 min at 4 °C, cells were stained with a fluorescein isothiocyanate (FITC)-conjugated anti-BrdU antibody for 30 min, counterstained with 7-AAD, and immediately analysed on a FACSCalibur instrument (BD Biosciences). Relative cell number (Figures 3C,D) was determined by counting cells in ImageJ (Schneider et al., 2012) on representative phase-contrast images acquired 2 and 7 days after transfection, using four independent biological donors. Counts were expressed relative to the untreated control of the same experiment.

### 2.15 Ubiquitin proteome profiler

A Human Ubiquitin Array (R&D Systems, Abingdon, United Kingdom) was performed according to the manufacturer’s instructions on lysates of scrambled-control and TRIM28-siRNA-transfected KCs processed in parallel within the same experiment. Each target is represented by duplicate spots on the array. These are technical replicates and were averaged to a single value per array. Two independent arrays were run, each on lysates from a separate KC preparation, so that each plotted value represents one biological replicate. Spot intensities were quantified densitometrically in ImageJ (Schneider et al., 2012) and expressed relative to the paired scrambled control on the same array. Given the small number of biological replicates, no statistical test was applied and the result is reported descriptively.

### 2.16 Statistics

Statistical analyses were performed with GraphPad Prism version 10.0.3 (GraphPad Software, San Diego, CA, United States). Normality was assessed with the Kolmogorov–Smirnov test. A p-value <0.05 was considered statistically significant, and data are expressed as mean ± standard deviation (SD). Independent biological replicates, that is independent KC preparations, independent skin equivalents or independent skin donors, were used as the statistical unit throughout. Multiple images, sections or nuclei from the same sample were averaged to the mean per biological replicate before analysis. Where experimental conditions were derived from the same donor preparation, each replicate was normalised to its own untreated control from that same preparation. The resulting measurements were donor-matched and analysed accordingly. Two matched groups were compared by paired two-tailed t-test. Repeated-measures one-way ANOVA was used for matched designs with more than two conditions, followed by Dunnett’s multiple-comparisons test where the siRNA conditions were compared against their common control, or by Tukey’s multiple-comparisons test where all conditions were compared with one another. Where two conditions were compared at several time points, a paired two-tailed t-test was performed separately for each time point, with Holm–Šídák correction for multiple comparisons across time points. Where the number of biological replicates was too small to support inference, results are reported descriptively without a statistical test. The test applied, the statistical unit and the number of biological replicates are stated for each quantitative panel in the corresponding figure legend.

## 3 Results

### 3.1 Transcriptional differences between KCs differentiated in monolayer cultures and skin models

**FIGURE 1:**
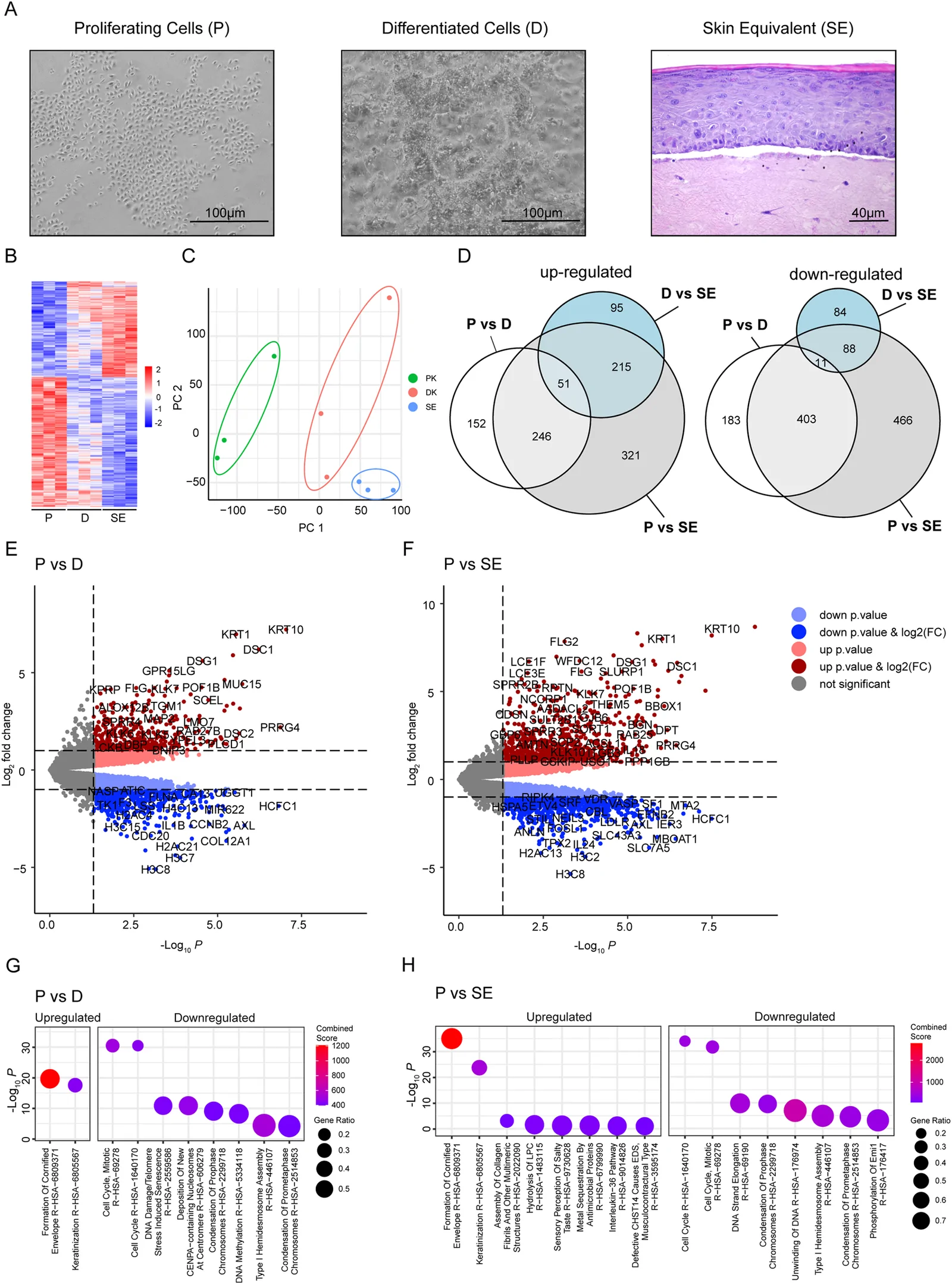
Transcriptional differences between KCs differentiated in monolayer culture and in skin equivalents. **(A)** Representative morphology of the three conditions analysed: undifferentiated, proliferating KCs (P) and differentiated KCs (D) in monolayer culture (phase-contrast micrographs), and a fully stratified skin equivalent (SE; hematoxylin–eosin-stained section). Scale bars, 100 μm and 40 µm, respectively. **(B)** Heatmap with hierarchical clustering of differentially expressed genes (DEGs) across the P, D, and SE conditions (n = 3 biological replicates per condition); colour indicates row-scaled expression (z-score). **(C)** Principal component analysis (PCA) of the normalised expression data, showing separation of proliferating KCs (PK, green), differentiated KCs (DK, red), and skin equivalents (SE, blue). **(D)** Area-proportional Euler diagrams showing the numbers and overlaps of significantly upregulated (left) and downregulated (right) genes for the three pairwise comparisons (P vs. D, P vs. SE, and D vs. SE; FDR <0.05, |logFC| ≥ 1). **(E,F)** Volcano plots for P vs. D **(E)** and P vs. SE **(F)**; the x-axis shows −log10 (p-value) and the y-axis the log2 fold change. Selected genes are labelled and coloured by significance category (grey, not significant; up- or downregulated by p-value alone or by both p-value and log2 fold change, as indicated in the key). DEGs were defined as |logFC| ≥ 1 with FDR <0.05 **(G,H)** Reactome pathway enrichment (dot plots) of the upregulated (left) and downregulated (right) gene lists for P vs. D **(G)** and P vs. SE **(H)**, ranked by the Enrichr combined score. Dot size indicates the gene ratio, dot colour the combined score, and the y-axis the −log10 (p-value).

To investigate transcriptional changes associated with primary human KC differentiation, we compared undifferentiated proliferating KCs (P), KCs differentiated in monolayer culture (D), and fully stratified organotypic skin equivalents (SE) (Figure 1A). Hierarchical clustering of differentially expressed genes (DEGs; Figure 1B) and principal component analysis (PCA; Figure 1C) revealed clearly distinct transcriptional profiles across the three conditions. Relative to proliferating KCs, differentiation in skin equivalents resulted in the upregulation of 833 mRNAs and the downregulation of 968 mRNAs, whereas monolayer differentiation resulted in the upregulation of 449 mRNAs and the downregulation of 597 mRNAs (Figure 1D). In both differentiation models, most downregulated genes were associated with cell-cycle progression (Figures 1E–H). By contrast, late differentiation-associated transcripts, including *FLG2*, *WFDC12*, *SLURP1*, and members of the *LCE* and *SPRR* gene families, were more prominently induced in skin equivalents than in monolayer cultures (Figures 1E,F). Moreover, genes involved in collagen assembly and fibril formation were induced preferentially in skin equivalents (Figure 1H). These findings indicate that organotypic skin equivalents more closely recapitulate the transcriptional programme of *in vivo* KC maturation than monolayer differentiation and suggest that the three-dimensional collagen-rich environment contributes to the activation of extracellular-matrix-associated processes.

### 3.2 Identification of transcription-factor activity in differentially expressed genes during KC differentiation

**FIGURE 2:**
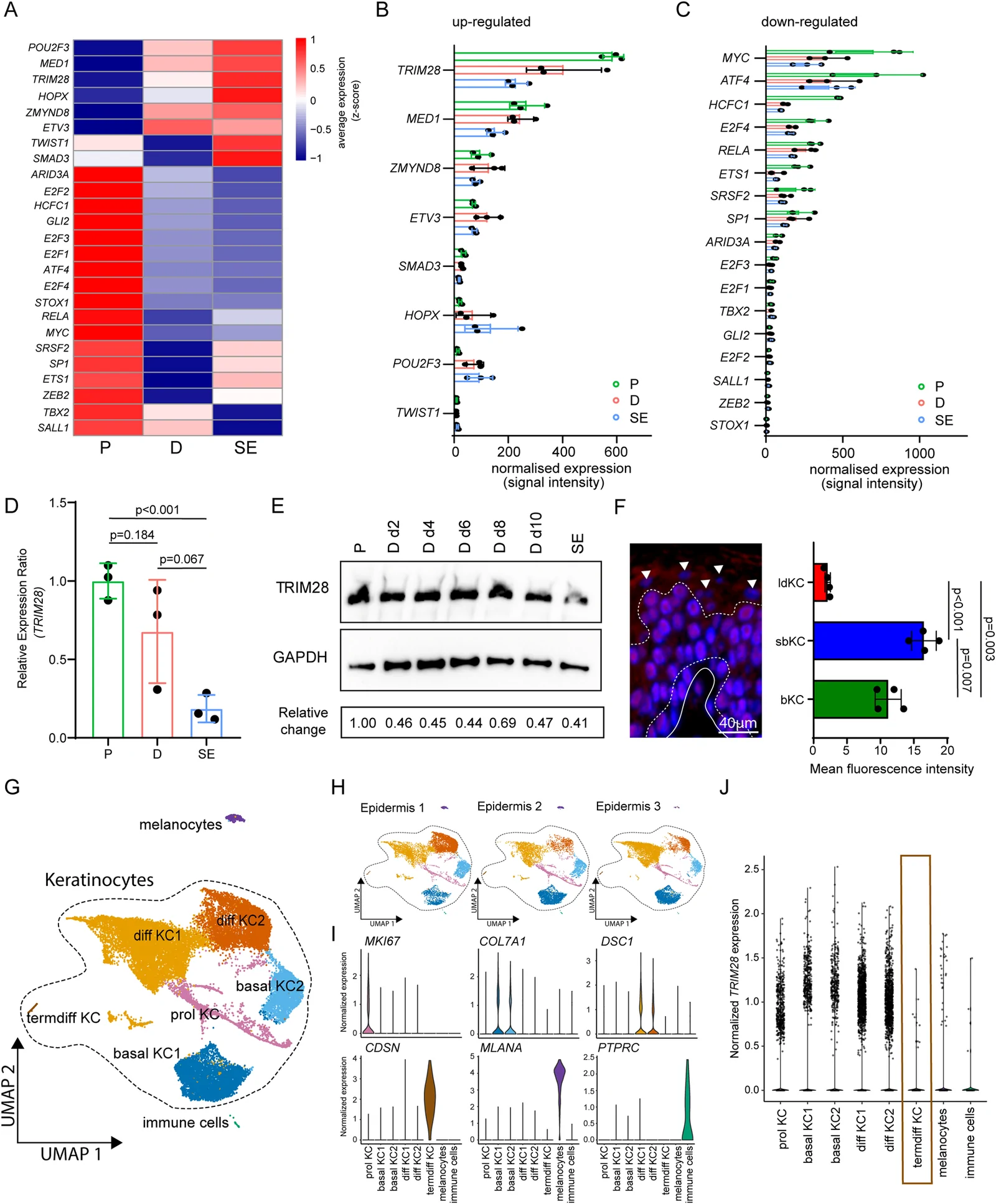
TRIM28 is expressed throughout the living epidermis and is lost in terminally differentiated KCs. **(A)** Heatmap of the inferred activity of the 25 regulators with the highest variance across the P, D, and SE conditions, averaged per condition and row-scaled. Activity was inferred with decoupleR from the expression of CollecTRI-annotated target genes and therefore reflects target-gene behaviour, not TRIM28 transcript abundance or protein level. **(B,C)** Normalised microarray expression (signal intensity) of the regulators with increased **(B)** and decreased **(C)** inferred activity during KC differentiation, shown for P (green), D (red), and SE (blue); bars represent mean ± SD with biological replicates (n = 3). Note that TRIM28 transcript levels are highest in P while its inferred activity is highest in SE, indicating regulation beyond transcript abundance. **(D)** qPCR validation of TRIM28 mRNA in P, D, and SE, expressed as the relative expression ratio (B2M as reference gene). Bars show mean ± SD with individual values (n = 3 biological replicates). Because all three conditions were derived from the same KC preparations, they were compared by paired two-tailed t-test; p-values are indicated. **(E)** Western blot of TRIM28 and GAPDH (loading control) in proliferating KCs (P), KCs differentiated in monolayer for 2–10 days (D d2 – D d10) and skin equivalents (SE); the relative change below the blot is the densitometric TRIM28/GAPDH ratio of the blot shown, normalised to P. These values describe the displayed blot only and are not derived from independent biological replicates, and no statistical test was applied. **(F)** Immunofluorescence staining of TRIM28 (red) with DAPI nuclear counterstain (blue) in human skin; arrowheads indicate the loss of nuclear TRIM28 in the outermost living epidermal layer, dotted lines delineate the epidermal layers and the solid line the dermal–epidermal junction. Scale bar, 40 µm. Right: quantification of nuclear TRIM28 mean fluorescence intensity in basal (bKC), suprabasal (sbKC) and last living differentiated (ldKC) KCs. For each of four independent human skin donors, the mean intensity of all nuclei assigned to a given layer was calculated. Donor means are plotted as individual points, with bars representing mean ± SD (n = 4 donors). Because every donor contributed all three layers, the layers were compared by repeated-measures one-way ANOVA with Tukey’s multiple-comparisons test; p-values are indicated. **(G)** UMAP projection of the single-cell RNA-sequencing dataset of healthy human epidermis (three donors, RPCA-integrated, clustering resolution 0.4), showing the annotated populations (proliferating, two basal, two differentiated and terminally differentiated KCs; melanocytes; immune cells). **(H)** The same UMAP projection split by donor (Epidermis 1–3). **(I)** Violin plots of representative marker genes used for cluster annotation (MKI67, proliferation; COL7A1, basal; DSC1 and CDSN, differentiation; MLANA, melanocytes; PTPRC, immune cells). **(J)** Log-normalised TRIM28 expression across the epidermal clusters; the box highlights the terminally differentiated KC cluster, in which TRIM28 is markedly reduced.

To identify potential regulators of epidermal differentiation, we inferred regulator activity from the expression of documented target genes using the decoupleR package together with the CollecTRI regulon collection (Müller-Dott et al., 2023). The inferred activities of the 25 TFs with the highest variance across proliferating KCs, monolayer-differentiated KCs, and skin equivalents are shown in Figure 2A. Seventeen TFs showed significantly reduced inferred activity in both differentiation models, whereas eight showed strongly increased inferred activity during differentiation, with the highest activity scores observed in skin equivalents (Figure 2A).

Because this approach infers TF activity from the expression of known target genes rather than directly measuring TF binding or protein abundance, we next examined the expression levels of the identified TFs to support the computational predictions (Figures 2B,C). Among the TFs highlighted by this analysis, TRIM28 showed the highest mRNA expression in proliferating KCs (Figure 2B), and its expression decreased during monolayer differentiation, with an even more pronounced reduction in skin equivalents (Figure 2B). This apparent discrepancy between mRNA expression and inferred activity (high transcript abundance but low inferred activity in proliferating KCs, and reduced transcript abundance but high inferred activity in skin equivalents) suggests that TRIM28 function may be regulated beyond transcript abundance, for example, through post-translational modification, phosphorylation, or differential protein-protein interactions that modulate its repressive activity independently of transcript levels.

To validate these transcriptomic findings, we performed qPCR and Western blot analyses. qPCR confirmed significantly lower *TRIM28* mRNA levels during differentiation in skin equivalents (Figure 2D). In contrast, TRIM28 protein levels were highest in proliferating KCs but remained largely stable during monolayer differentiation and in skin equivalents (Figure 2E). To examine TRIM28 protein distribution across epidermal layers, we performed immunofluorescence staining of human skin and quantified nuclear TRIM28 signal intensity using ImageJ (Figure 2F). TRIM28 was abundantly expressed in the nuclei of basal and suprabasal KCs, whereas nuclear staining was strongly reduced in the outermost living epidermal layer of the epidermis (Figure 2F). Of note, this layer retains cellular organelles, indicating that the loss of TRIM28 staining is not simply a consequence of nuclear degradation. To assess TRIM28 expression in the epidermis at single-cell resolution, we re-analysed a scRNA-seq dataset of healthy human epidermis (Figures 2G–J). Unsupervised clustering identified six KC populations together with melanocytes and immune cells (Figure 2G), which were present in all three donors (Figure 2H), and cluster identities were assigned using canonical marker genes (Figure 2I). Violin plots confirmed that *TRIM28* mRNA was abundant in basal and suprabasal KCs but markedly reduced in terminally differentiated cells (Figure 2J). Together, these results indicate that TRIM28 is dynamically regulated during KC differentiation and is selectively lost from the terminally differentiated epidermal compartment.

### 3.3 TRIM28 is required for keratinocyte survival and normal epidermal development

**FIGURE 3:**
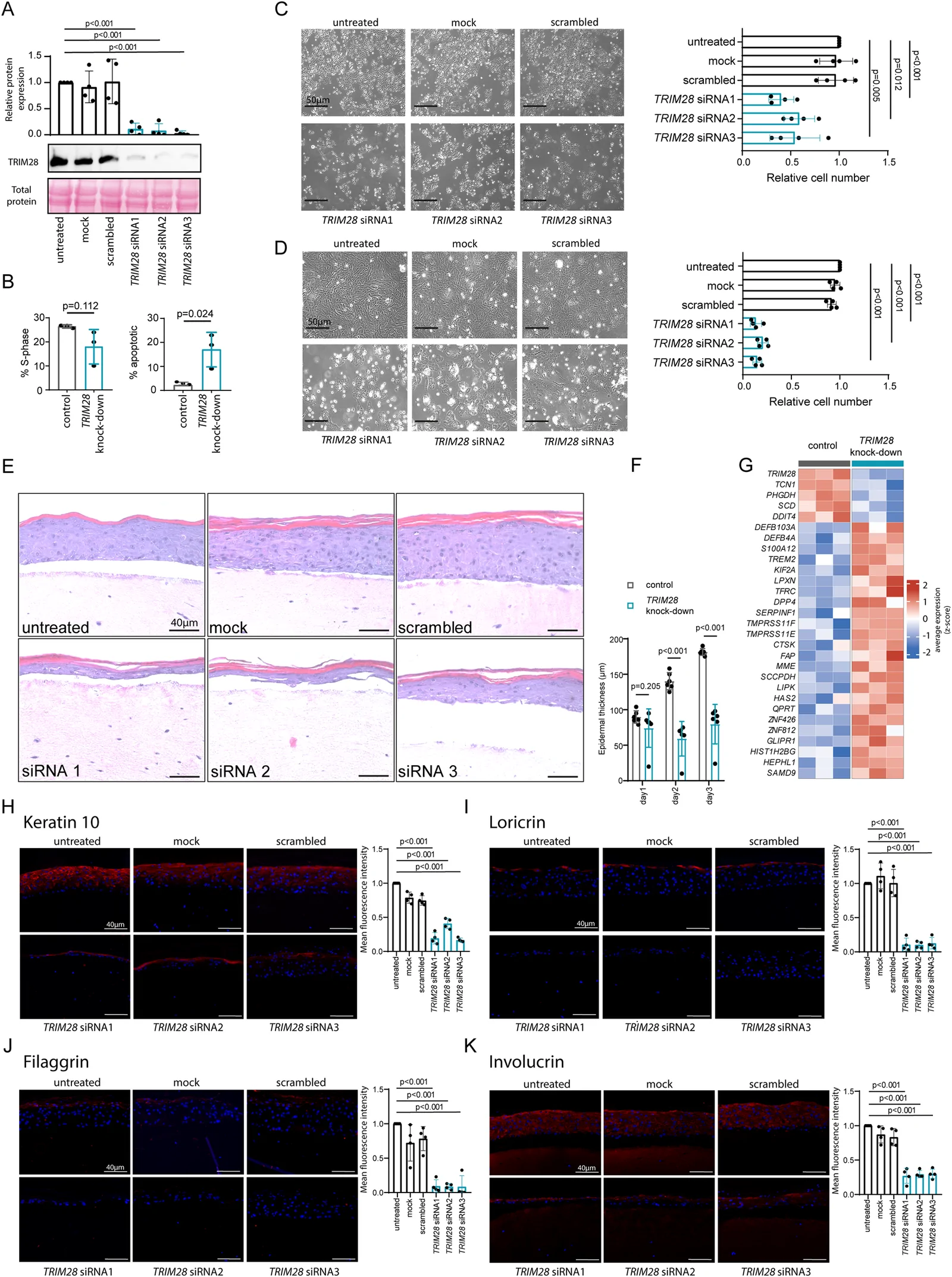
TRIM28 is required for KC proliferation, survival, and epidermal expansion. **(A)** Knockdown of TRIM28 in primary human KCs by three independent TRIM28-specific siRNAs (siRNA1–3), compared with untreated, mock-transfected and scrambled-control cells. Representative Western blot with total protein staining as loading control, and densitometric quantification of relative TRIM28 protein expression (mean ± SD with individual values, n = 4 biological replicates). Statistical comparison was performed by repeated-measures one-way ANOVA with Dunnett’s multiple-comparisons test against the untreated control; p-values are indicated. **(B)** BrdU/7-AAD flow-cytometric analysis of control and TRIM28-knockdown KCs, showing the proportion of cells in S phase (left) and of apoptotic cells (right). Bars show mean ± SD with individual values (n = 3 biological replicates). Statistical comparison was performed by paired two-tailed t-test. p-values are indicated. **(C,D)** Representative phase-contrast images of KCs 2 days **(C)** and 7 days **(D)** after transfection with the indicated siRNAs; relative cell numbers were quantified from representative images and expressed relative to the untreated control (right; mean ± SD with individual values, n = 4 independent biological donors). Statistical comparison was performed by repeated-measures one-way ANOVA with Dunnett’s multiple-comparisons test against the untreated control; p-values are indicated. TRIM28-deficient cultures show impaired expansion at day 2 and, by day 7, impaired growth and increased cell death. Scale bars, 50 µm. **(E)** Hematoxylin-eosin-stained sections of skin equivalents generated from untreated, mock, scrambled and TRIM28-siRNA (siRNA1-3) transfected KCs, 7 days after exposure to the air–liquid interface. Scale bars, 40 µm. **(F)** Epidermal thickness (µm) of control and TRIM28-knockdown skin equivalents on days 1–3 after exposure to the air–liquid interface. Bars show mean ± SD with individual values (n = 6 independent skin equivalents per group). Control and TRIM28-knockdown equivalents at each time point were generated from the same KC preparation. The two conditions were compared by paired two-tailed t-test at each time point, with Holm–Šídák correction for multiple comparisons across time points; p-values are indicated. **(G)** Heatmap of a selection of transcripts differentially expressed between scrambled-control and TRIM28-knockdown skin equivalents (paired analysis, n = 3 donors per group; FDR <0.05 and |logFC| ≥ 1). Colour indicates row-scaled average expression (z-score). **(H–K)** Immunofluorescence staining for keratin 10 **(H)**, loricrin **(I)**, filaggrin **(J)** and involucrin **(K)** (all red) with DAPI nuclear counterstain (blue) in skin equivalents 7 days after exposure to the air–liquid interface, with quantification as mean fluorescence intensity relative to untreated controls (mean ± SD with individual values, n = 4 independent skin equivalents). All sections of one skin equivalent were averaged to the mean value per skin equivalent. Statistical comparison was performed by repeated-measures one-way ANOVA with Dunnett’s multiple-comparisons test against the untreated control; p-values are indicated. Scale bars, 40 µm.

To investigate the functional role of TRIM28 in epidermal development and barrier formation, we knocked down TRIM28 in primary human KCs using three independent TRIM28-specific siRNAs, each tested separately, and subsequently established organotypic skin models. All three siRNAs achieved >95% knockdown efficiency, as confirmed by Western blot analysis (Figure 3A). Two days after transfection, TRIM28-deficient KCs showed reduced S-phase entry, as assessed by BrdU incorporation, although this decrease did not reach statistical significance (Figure 3B). Total cell numbers, however, were significantly lower than in scrambled-control cultures, indicating impaired proliferative expansion (Figure 3C). Whereas control KCs reached full confluence and underwent differentiation by day 7, TRIM28-deficient KCs displayed impaired growth and increased cell death (Figure 3D), which was corroborated by cell-cycle analysis showing a significant increase in the proportion of apoptotic cells at day 7 (Figure 3B).

Skin equivalents were generated from both control and TRIM28-knockdown cells. Morphologically, TRIM28-deficient skin equivalents underwent stratification but were consistently and significantly thinner across all three siRNA conditions (Figures 3E,F). Although epidermal thickness was comparable on day 1 after exposure to the air–liquid interface, only control models thickened progressively over time, whereas TRIM28-knockdown models remained thin and failed to expand their suprabasal compartment (Figure 3F). Although these skin models still formed a cornified layer, several differentiation-associated marker proteins were significantly reduced in TRIM28-deficient skin equivalents (Figures 3H–K), suggesting an additional differentiation defect.

To determine whether these morphological and differentiation-related changes were accompanied by broader transcriptional reprogramming, we performed global gene-expression profiling of TRIM28-deficient skin equivalents. Unexpectedly, only 33 genes were significantly upregulated and 5 were downregulated relative to controls (Figure 3G). Functional annotation of the upregulated genes revealed enrichment for immune and antimicrobial-defence transcripts, for example, *S100A12* and *DEFB4A*, and for extracellular-matrix remodelling, for example, *SERPINF1* and *CTSK*. Because only a limited number of regulated genes were associated with developmental processes, these transcriptional changes alone are unlikely to account for the pronounced morphological phenotype. These data therefore suggest that TRIM28 has a limited role in the transcriptional regulation of epidermal gene expression in this setting and that post-transcriptional mechanisms may contribute substantially to the effects observed in TRIM28-knockdown skin equivalents.

### 3.4 TRIM28 deficiency is associated with p53 accumulation, apoptosis, and reduced p53 ubiquitination

**FIGURE 4:**
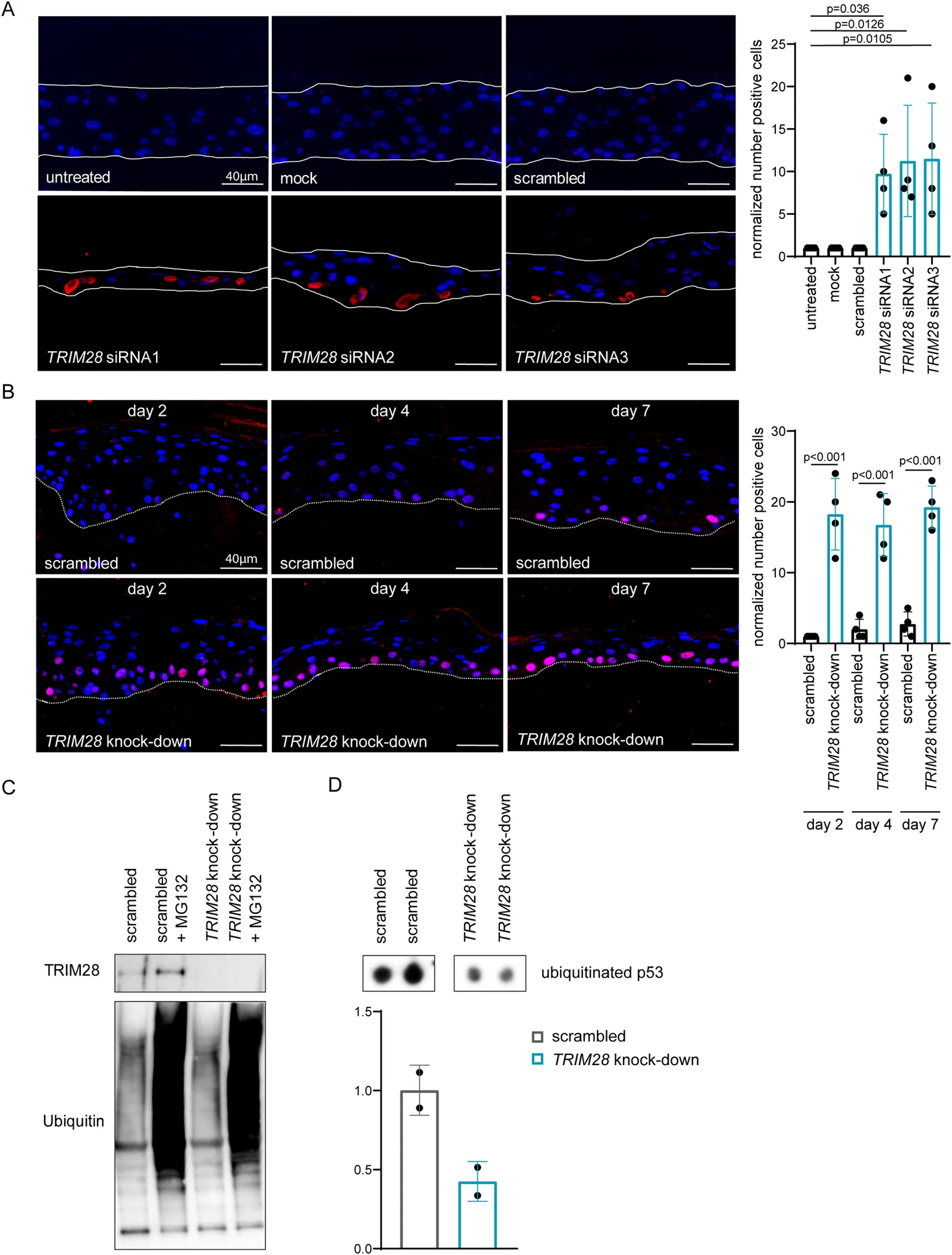
TRIM28 deficiency is associated with p53 accumulation, basal-KC apoptosis and a reduced ubiquitinated-p53 signal. **(A)** Immunofluorescence staining of active caspase-3 (red) with DAPI nuclear counterstain (blue) in skin equivalents generated from untreated, mock, scrambled and TRIM28-siRNA (siRNA1–3) transfected KCs, 7 days after exposure to the air-liquid interface. White lines delineate the epidermis; active caspase-3 is confined to the basal layer of TRIM28-deficient models. Right: number of active caspase-3-positive cells normalised to untreated controls (mean ± SD with individual values, n = 4 independent skin equivalents). All sections of one skin equivalent were averaged to the mean value per skin equivalent. Statistical comparison was performed by repeated-measures one-way ANOVA with Dunnett’s multiple-comparisons test against the untreated control; p-values are indicated. Scale bars, 40 µm. **(B)** Immunofluorescence staining of p53 (red) with DAPI nuclear counterstain (blue) in scrambled-control (upper) and TRIM28-knockdown (lower) skin equivalents at days 2, 4 and 7 after exposure to the air-liquid interface; dotted lines indicate the dermal–epidermal junction and nuclear p53 appears magenta. Right: number of p53-positive cells normalised to the corresponding scrambled control (mean ± SD with individual values, n = 4 independent skin equivalents). Because control and TRIM28-knockdown equivalents at each time point were generated from the same KC preparation, the two conditions were compared by paired two-tailed t-test at each time point, with Holm–Šídák correction for multiple comparisons across the three time points; p-values are indicated. Scale bars, 40 µm. **(C)** Western blot of TRIM28 and total ubiquitin in scrambled-control and TRIM28-knockdown KCs, with or without the proteasome inhibitor MG132; global protein ubiquitination is unchanged by TRIM28 knockdown. Representative of three independent experiments. **(D)** Human ubiquitin antibody array showing ubiquitinated p53 in scrambled-control and TRIM28-knockdown KCs. Each target is represented by duplicate spots on the array. These are technical replicates and were averaged to one value per array. Two independent arrays were run, each on lysates from a separate KC preparation, and the two plotted points therefore represent two biological replicates, each normalised to its paired scrambled control on the same array. Because only two biological replicates are available, no statistical test was applied and no p-value is reported. The arrays show a signal consistent with reduced ubiquitinated p53 after TRIM28 knockdown and should be regarded as representative rather than quantitative.

We first examined whether the reduced epidermal thickness observed after TRIM28 knockdown was associated with increased cell death. Staining for active caspase-3, a marker of apoptosis, revealed prominent labelling in basal KCs of TRIM28-deficient skin equivalents (Figure 4A). In control skin equivalents, p53 immunostaining was minimal or undetectable at days 2 and 4, with focal accumulation in a subset of basal cells only by day 7 (Figure 4B, upper panel). In contrast, TRIM28-deficient skin equivalents displayed robust and widespread p53 staining throughout the basal and several suprabasal layers at all time points analysed (Figure 4B, lower panel).

To determine whether p53 accumulation was accompanied by impaired ubiquitination, we assessed both global and p53-specific ubiquitination. TRIM28 knockdown did not detectably alter global protein ubiquitination, either in untreated KCs or in KCs treated with the proteasome inhibitor MG132 (Figure 4C). By contrast, the ubiquitinated-p53 signal on the array was lower in TRIM28-deficient cells in both of the two independent arrays analysed (Figure 4D). Because only two biological replicates were available, this observation is descriptive and was not tested statistically. These findings suggest that TRIM28 contributes to p53 turnover in basal KCs and that impaired p53 ubiquitination may promote p53 accumulation and apoptosis in the basal compartment, thereby limiting epidermal expansion.

## 4 Discussion

In this study, we identified the transcriptional repressor and ubiquitin/SUMO E3 ligase TRIM28 as a candidate regulator of epidermal homeostasis. By integrating transcriptomic profiling of two complementary differentiation models with single-cell expression data and loss-of-function experiments in organotypic skin, we show that TRIM28 is expressed throughout the living epidermal layers but is selectively lost from KCs of the outermost living layer. Its depletion in proliferating KCs was associated with reduced p53 ubiquitination, which was accompanied by p53 accumulation, apoptosis of basal KCs, ultimately resulting in a markedly thinned epidermis. Together, these observations support a model in which TRIM28 may help maintain basal KC survival by restraining p53 accumulation, thereby permitting orderly epidermal stratification and differentiation.

A central and initially counterintuitive observation of our study concerns the regulation of TRIM28 itself. Although *TRIM28* mRNA decreased progressively during differentiation (most strongly in the 3D skin models) TRIM28 protein levels remained largely stable in KC cultures, and immunostaining localised the protein to basal and suprabasal KCs, with a marked loss confined to the outermost, terminally differentiated layer. This discordance between transcript and protein is consistent with post-transcriptional stabilisation of TRIM28 in the living epidermis and suggests that the biologically relevant event may be not only the gradual decline in transcription but the abrupt loss of the protein at the final stage of differentiation. Because p53 activity is known to increase during KC differentiation even as p53 abundance decreases (Einspahr et al., 1999; Cai et al., 2012), the spatial restriction of TRIM28 may help explain how the epidermis balances these opposing requirements: TRIM28 may limit p53 accumulation in the proliferative basal compartment, where excessive p53 activity could promote apoptosis, while its loss in the uppermost layer may permit p53-associated terminal events of cornification and shedding.

Our experimental design complements earlier work on the dynamics of KC differentiation. Janich et al. showed that core circadian-clock genes peak in a phased manner in human epidermal stem cells and their differentiated progeny (Janich et al., 2013), whereas Klingenberg et al. compared engineered skin after *in vivo* grafting with *in vitro* maturation and normal human skin to define gene programmes associated with tissue maturation (Klingenberg et al., 2010). While these and related studies focused either on early differentiation *in vitro* or on long-term changes in whole-skin samples, we directly compared proliferating populations with fully differentiated KCs in both monolayer and organotypic culture. The principal advantage of organotypic systems over monolayer culture is their capacity to reproduce the three-dimensional, stratified architecture in which KCs normally reside. Consistent with this, the skin equivalents more closely recapitulated the *in vivo* differentiation programme, including the induction of late-differentiation and ECM-assembly genes. Such models nonetheless reproduce only part of normal skin organisation and are constrained in size by the inefficient diffusion of nutrients from the culture medium (Oh et al., 2013), a limitation that should be considered when extrapolating to intact human skin.

Most TFs predicted to drive late differentiation in previous analyses, such as TAp63 and Notch effectors (Koh et al., 2015; Kouwenhoven et al., 2015), are sequence-specific transcriptional regulators. By contrast, our TF activity inference using skin-model-specific transcripts highlighted TRIM28/KAP1, which was originally described as a universal corepressor for KRAB–zinc-finger proteins (Friedman et al., 1996) and is therefore expected to influence large numbers of genes indirectly. It was thus notable that knockdown of TRIM28 in skin equivalents altered the expression of only a small set of genes, predominantly immune- and antimicrobial-defence transcripts and proteases, rather than the broad developmental signature that a classical transcriptional repressor might be expected to derepress. This limited transcriptional response, despite a striking morphological phenotype, suggests that TRIM28 may act in this context mainly through post-translational mechanisms, including E3-ligase-related functions, rather than through broad transcriptional repression.

Mechanistically, our data place TRIM28 within the TRIM28-MDM2-p53 axis. TRIM28 has been reported to cooperate with MDM2 to promote the ubiquitination and proteasomal degradation of p53, and MAGE–TRIM28 complexes have been shown to target p53 for proteasome-dependent degradation (Wang et al., 2005; Doyle et al., 2010), and we found that TRIM28 depletion was accompanied by a lower ubiquitinated-p53 array signal without a measurable change in the global ubiquitination landscape, even in the presence of MG132. It should be noted that our data do not distinguish between direct TRIM28-mediated ubiquitination of p53 and an indirect effect via support of MDM2 activity. Resolving this mechanistic question is an important direction for future work. The resulting accumulation of p53 was accompanied by apoptosis, precisely where proliferating, p53-sensitive progenitors reside. Histologically, TRIM28-deficient skin equivalents retained a morphologically differentiated stratum corneum despite reduced basal-layer cellularity, suggesting that p53 accumulation may thin the proliferative compartment without completely preventing stratum corneum formation, although differentiation-associated factors were also affected. These findings are consistent with genetic evidence in mice, in which epidermis-specific deletion of *Mdm2*, the principal negative regulator of p53, causes epidermal thinning, impaired wound healing, and stem-cell loss (Gannon et al., 2011), and in which sustained p53 activation drives skin ageing and stem-cell depletion (Kim et al., 2014). They also align with the observation that inactivation of p53 in human KCs deregulates differentiation and shedding (Freije et al., 2014), underscoring that epidermal homeostasis requires p53 activity to be maintained within a narrow window. Our results suggest that TRIM28 contributes to this balance, at least in part, through regulation of p53 ubiquitination.

We considered alternative explanations for the apoptotic phenotype. TRIM28 has been reported to ubiquitinate the anti-apoptotic protein BCL2A1 (Lionnard et al., 2019). However, in that setting TRIM28 promotes BCL2A1 degradation, so its loss would be expected to stabilise BCL2A1 and oppose, rather than promote, apoptosis. This makes the BCL2A1 route an unlikely driver of the basal-cell death observed here and is therefore consistent with, although it does not establish, a p53-associated mechanism. More broadly, TRIM28 has context-dependent and sometimes opposing roles in cancer: it is frequently overexpressed and behaves as an oncogenic factor, for example, by promoting p53 degradation and by stabilising pro-metastatic factors such as TWIST1 (Liu et al., 2013; Wei et al., 2016; Czerwińska et al., 2017), yet it can also restrain proliferation and enforce senescence in other settings (Santos and Gil, 2014; Miles et al., 2017; Li et al., 2024). This duality likely reflects tissue- and context-specific substrate availability and again points to a prominent post-transcriptional mode of action, in line with the limited transcriptional response we observed in KCs.

Several limitations temper our conclusions. Knockdown was achieved with transient siRNA, so we cannot exclude residual TRIM28 activity or off-target contributions, although the concordant phenotypes obtained with three independent siRNAs mitigate this concern. The organotypic model, while more physiological than monolayer culture, lacks immune cells, vasculature, and adnexal structures, and our mechanistic readouts were obtained largely at the protein and histological levels rather than through genetic epistasis with p53. Direct demonstration that p53 is required for the phenotype, for example, through concomitant p53 silencing or pharmacological inhibition in TRIM28-deficient skin equivalents, would further strengthen the causal link and represents an important next step. Rescue by re-expression of TRIM28 would provide a further specificity control, but stable re-expression in primary KCs requires antibiotic selection over several passages, and high-passage KCs no longer form morphologically normal skin equivalents. Inducible systems or immortalised KC lines, as well as stable CRISPR/Cas9-mediated knockout, are therefore appropriate approaches for future work. Finally, whether the spatial loss of TRIM28 in the uppermost epidermal layer is a cause or a consequence of terminal differentiation remains to be established.

From a translational perspective, our findings suggest that dysregulation of the TRIM28-p53 axis could contribute to disorders characterised by disturbed epidermal proliferation, differentiation, or barrier function, such as psoriasis, atopic dermatitis, and cutaneous squamous cell carcinoma. Indeed, re-analysis of published scRNA-seq datasets indicates downregulation of TRIM28 in KCs from lesional and non-lesional psoriasis samples and upregulation in squamous cell carcinomas *in situ* (Supplementary Figure S2). This initial analysis should be viewed as a first step toward a broader research programme aimed at defining how TRIM28-dependent regulation of p53 and epidermal homeostasis contributes to inflammatory and neoplastic skin disease.

Given the central role of TRIM28 in controlling proliferation, differentiation, and apoptosis, and its likely importance for homeostasis in other self-renewing tissues (Santos and Gil, 2014; Miles et al., 2017; Lu et al., 2020; Maghsoudloo et al., 2024), modulation of the TRIM28-p53 axis may represent an avenue worth exploring for these conditions. Defining the upstream signals that stabilise TRIM28 in the basal epidermis and trigger its removal during terminal differentiation will be essential before these observations can be translated into therapeutic strategies.

## 5 Conclusion

We investigated the role of TRIM28 in epidermal homeostasis and found that TRIM28 is expressed in KCs throughout the living epidermal layers but is lost from the outermost terminally differentiated compartment. SiRNA-mediated depletion of TRIM28 was associated with reduced p53 ubiquitination, p53 accumulation, increased apoptosis of basal KCs, and epidermal thinning. These findings identify TRIM28 as an important candidate regulator of KC survival and epidermal development and suggest that its contribution to p53 turnover may help support basal-cell maintenance and epidermal expansion.

## Data Availability

The datasets presented in this study can be found in online repositories. The names of the repository/repositories and accession number(s) can be found below: https://www.ncbi.nlm.nih.gov/geo/query/acc.cgi?acc=GSE145059, https://www.ncbi.nlm.nih.gov/geo/query/acc.cgi?acc=GSE334376.
