# Supplementary material for: TRIM28 contributes to epidermal homeostasis and is associated with altered p53 regulation
Source: Front Cell Dev Biol. 2026 Sep 8;14:1903169. doi: 10.3389/fcell.2026.1903169 (PMC13595995; doi:10.3389/fcell.2026.1903169)
Supplement: Supplementary file 1 [file Supplementaryfile1.docx]

Supplementary Material

# 1 Supplementary Data

This file contains Supplementary Figures S1 and S2 with their legends.

The R scripts used for microarray preprocessing and differential expression analysis and for single-cell RNA-sequencing processing are provided as separate .txt files in “Supplementary Material scripts.zip”.

# 2 Supplementary Figures

**Supplementary Figure S1. Quality control, doublet removal and donor integration of the human epidermal single-cell RNA-sequencing dataset.**

**(A)** Violin plots of UMI counts (nCount RNA), detected genes (nFeature RNA) and mitochondrial transcript content (percent mt) per donor (Epidermis 1–3) before filtering. **(B)** UMI counts versus detected genes per cell before filtering, coloured by mitochondrial content. **(C)** The same quality-control metrics after applying the common cutoffs (800–4,000 detected genes, 2,000–25,000 UMIs, <10% mitochondrial transcripts) and doublet removal. **(D)** UMI counts versus detected genes per cell after filtering, coloured by the scDblFinder classification into singlets and predicted doublets; doublets were excluded from all downstream analyses. **(E)** Standard deviation (left) and cumulative variance explained (right) per principal component; the dashed line marks the 30 principal components used for clustering and visualisation. **(F)** UMAP projections coloured by donor before (left) and after (right) reciprocal PCA integration, showing that donor-specific separation is resolved by integration.

**Supplementary Figure S2. TRIM28 expression in keratinocytes of published single-cell RNA-sequencing datasets of inflammatory and neoplastic human skin.**

Publicly available datasets were re-processed with the same workflow as the healthy-epidermis dataset and keratinocytes were annotated as proliferating (pKC), basal (bKC), suprabasal (sbKC) and differentiated (dKC). **(A–C)** UMAP projections of the annotated keratinocyte populations for **(A)** psoriasis (Ma et al., 2023), **(B)** atopic dermatitis (Bangert et al., 2021) and **(C)** keratinocyte tumours (Schütz et al., 2023; Zou et al., 2023; Ji et al., 2020). **(D–F)** Dot plots of the marker genes used for annotation in the corresponding datasets; dot size indicates the percentage of expressing cells and colour the scaled average expression. **(G–I)** Dot plots of TRIM28 expression per keratinocyte population and condition: **(G)** lesional, non-lesional psoriatic and healthy skin; **(H)** atopic dermatitis (AD) and healthy skin; and **(I)** cutaneous squamous cell carcinoma (cSCC), squamous cell carcinoma in situ (SCCIS), actinic keratosis (AK) and healthy skin.
